# Past and potential future population dynamics of three grouse species using ecological and whole genome coalescent modeling

**DOI:** 10.1002/ece3.4163

**Published:** 2018-05-29

**Authors:** Radoslav Kozma, Mette Lillie, Blas M. Benito, Jens‐Christian Svenning, Jacob Höglund

**Affiliations:** ^1^ Department of Ecology and Genetics Evolutionary Biology Centre Uppsala University Uppsala Sweden; ^2^ Department of Biological and Environmental Sciences University of Gothenburg Göteborg Sweden; ^3^ Department of Bioscience Section for Ecoinformatics and Biodiversity University of Aarhus Aarhus C Denmark

**Keywords:** climate change, demographic history, pairwise sequentially Markovian coalescent, Pleistocene, species distribution modeling, Tetraoninae

## Abstract

Studying demographic history of species provides insight into how the past has shaped the current levels of overall biodiversity and genetic composition of species, but also how these species may react to future perturbations. Here we investigated the demographic history of the willow grouse (*Lagopus lagopus*), rock ptarmigan (*Lagopus muta*), and black grouse (*Tetrao tetrix*) through the Late Pleistocene using two complementary methods and whole genome data. Species distribution modeling (SDM) allowed us to estimate the total range size during the Last Interglacial (LIG) and Last Glacial Maximum (LGM) as well as to indicate potential population subdivisions. Pairwise Sequentially Markovian Coalescent (PSMC) allowed us to assess fluctuations in effective population size across the same period. Additionally, we used SDM to forecast the effect of future climate change on the three species over the next 50 years. We found that SDM predicts the largest range size for the cold‐adapted willow grouse and rock ptarmigan during the LGM. PSMC captured intraspecific population dynamics within the last glacial period, such that the willow grouse and rock ptarmigan showed multiple bottlenecks signifying recolonization events following the termination of the LGM. We also see signals of population subdivision during the last glacial period in the black grouse, but more data are needed to strengthen this hypothesis. All three species are likely to experience range contractions under future warming, with the strongest effect on willow grouse and rock ptarmigan due to their limited potential for northward expansion. Overall, by combining these two modeling approaches, we have provided a multifaceted examination of the biogeography of these species and how they have responded to climate change in the past. These results help us understand how cold‐adapted species may respond to future climate changes.

## 1 INTRODUCTION

Advances within the field of biogeography in the recent past are providing novel insights into how the Earth’s past climate affected the distribution of organisms (Alvarado‐Serrano & Knowles, 2014; Lawing & Polly, 2011; Peterson & Ammann, 2013; Svenning, Fløjgaard, Marske, Nógues‐Bravo, & Normand, 2011; Svenning, Normand, & Kageyama, 2008). Such knowledge is invaluable, as past population dynamics shape the species’ present distributions, giving rise to diversity patterns seen today and affecting the overall genetic composition of population (Metcalf et al., 2014; Pedersen, Sandel, & Svenning, 2014).

An integral part of investigating the past dynamics of organisms is the “species distribution modeling” method (hereafter SDM), a mostly correlative approach for computing habitat suitability maps based on the statistical relationships between presence records and environmental predictors (usually climate; Svenning et al., 2011). The projection of habitat suitability maps at different time (either past or future) allows the visualization and quantification of the changes in distributions through time and the testing of evolutionary and biogeographical hypotheses (Graham, Ron, Santos, Schneider, & Moritz, 2004). Complementing SDM, purely genetic (i.e., haplotype network) as well as coalescent‐based methods (i.e., Bayesian skyline plots [BSP]) can be integrated to reveal a more detailed phylogeographic picture of the species’ past dynamics (Carstens & Richards, 2007; Porretta, Mastrantonio, Bellini, Somboon, & Urbanelli, 2012). These methods, however, can have limited sensitivity and as a result might not capture the full dynamics over the desired time scale (e.g., a very flat BSP with only a very recent population size change detected; Grant, 2015).

The advent of more affordable whole genome sequencing and enhancement of the coalescent framework (Marjoram & Tavaré, 2006) has now given rise to the possibility of extracting more information from fewer samples. One such method is the Pairwise Sequentially Markovian Coalescent model (PSMC; Li & Durbin, 2011). It allows for the tracking of species’ effective population size (*N*_e_) from 10 thousand years ago (kya) through to Early Pleistocene/Late Pliocene (~3 million years ago [Mya]) from the genome of just one individual (Hung et al., 2014; Nadachowska‐Brzyska, Li, Smeds, Zhang, & Ellegren, 2015; Zhao et al., 2013). It therefore the ideal tool to study whether the changes in range size through time, as determined by SDM, reflect the changes in population size. Combined, the two methods have the ability to reveal demographic history of species at an unprecedented level.

In a previous study, we utilized the PSMC method on three grouse species (willow grouse, *Lagopus lagopus*; rock ptarmigan, *Lagopus muta*; and black grouse, *Tetrao tetrix*) in order to study their reaction to climate change throughout the Pleistocene (Kozma, Melsted, Magnússon, & Höglund, 2016). Both willow grouse and rock ptarmigan are cold‐adapted species living all year round in the arctic tundra of the Holarctic (Höglund, Wang, Axelsson, & Quintela, 2013; Holder, Montgomerie, & Friesen, 1999). The black grouse has a more southern distribution, inhabiting boreal forest edges, bogs, and moorland throughout the Palearctic (Corrales & Höglund, 2012). This study detected three main periods of population change, coinciding with the (1) Early Pleistocene cooling (~2.5 Mya), (2) Mid‐Brunhes event (~430 kya), and (3) last glacial period (~110–12 kya). Counter‐intuitively, all three species reacted differently to cold temperatures within the last glacial period, leaving us to speculate that the PSMC was capturing lineage specific dynamics.

The current paper builds upon these previous results to explore the demographic history of the grouse in detail by combining PSMC and SDMs and subsequently test the concordance of their respective results. We focus on the Last Interglacial (LIG, ~130 kya), the Last Glacial Maximum (LGM, ~21 kya), the present distribution, and future scenarios of anthropogenic climate change (2050 and 2070). Here, in addition to adding SDM analyses, we have analyzed the genomes of individuals from different parts of the range as populations of the same species may have faced different climatic histories.

## 2 METHODS

### 2.1 Species distribution modeling

#### 2.1.1 Climate data

Datasets for 19 climatic variables at 10‐min resolution were downloaded from Bioclim (http://www.worldclim.org/bioclim) for the present conditions, LIG, LGM, projected year 2050 and projected year 2070. To avoid biasing the results using only one general circulation model for future projections, we averaged each variable over the “CCSM4,” “IPSL‐CM5A‐LR,” and “MP‐ESM‐LR” models using QGIS (QGIS Development Team 2015), assuming the representative concentration pathway (RCP) 4.5 greenhouse gas scenario. To reduce multicollinearity, we calculated the correlation coefficients among all the 19 climatic variables for the present time and plotted them in a dendrograph (Supporting Information Figure S1). For every cluster of variables that were highly correlated (variable distance ≤ 0.5), we chose the variable thought to have more influence on the distribution of the target species bases on their natural history. To further reduce any potential multicollinearity, variance inflation factors (VIF, Heiberger, 2016) were calculated for the selected variables. The final variables used in the modeling were: BIO5 (maximum temperature of the warmest month), BIO6 (minimum temperature of the coldest month), BIO12 (annual precipitation), BIO14 (precipitation of driest month), and BIO15 (precipitation seasonality—coefficient of variation).

#### 2.1.2 Occurrence data

We used two different presence datasets for all three species in order to take into account potential biases or data gaps: presence records downloaded from GBIF (http://www.gbif.org) and range maps downloaded from Birdlife international (Birdlife International 2014). GBIF data were filtered to remove duplicates, records without coordinates, or records with a special accuracy coarser than the grain size used in the variables (10 min of acr). To obtain the second presence dataset, we generated random points within the polygons, defining the range map of each species. Background points were created within the full map extent (Northern Hemisphere). In order to evaluate the SDMs, both “GBIF” and “range map” datasets were partitioned further into two geographic subsets. For the willow grouse and rock ptarmigan, all points west and east of the 15th meridian West were grouped into the “West” and “East” subset, respectively (corresponding to North America, Greenland, and Iceland in west vs. Eurasia in east, Supporting Information Figures S2 and S3). Geographical partitioning could not adequately predict the current range of the black grouse (see Section 3), so for this species, random partitioning was employed, with 75% of data used for training and 25% used for evaluation. Table 1 summarizes the various subsets used for modeling.

**Table 1:** The number of presence points/background points used to create the species distributions models for the “GBIF” and “Range map” dataset. In the case of the latter, these represent pseudo‐presence points

	GBIF	Range map
Willow grouse		
East	218/28,102	3,130/28,102
West	329/15,517	1,566/15,517
Rock ptarmigan		
East	46/28,102	1,838/28,102
West	208/15,517	1,881/15,517
Black grouse		
Train	200/20,996	1,594/20,937
Test	66/6,929	532/6,988

#### 2.1.3 Modeling and evaluation

For each species, the probability of presence at current climatic conditions was modeled using a weighted polynomial GLM (Lehmann, Overton, & Leathwick, 2002), trained on one subset (e.g., GBIF‐West), and evaluated on the other subset (e.g., GBIF‐East). Evaluation of the models was performed using the receiver operating characteristic (ROC) and its area under the curve (AUC), where AUC scores above 0.7 were deemed to indicate good model performance (Fielding & Bell, 1997; Swets, 1988). The best model was subsequently used to predict the species’ presence across other time periods. Finally, a 10% threshold probability was applied in order to obtain a binary “presence/absence” map, from which the species’ total range area was calculated.

Unless otherwise stated, all data preparation, modeling, and calculations were performed in R v3.2.2 R Core Team 2013; Hijmans, Phillips, Leathwick, & Elith, 2016) using the “dismo” (Hijmans et al., 2016) and “raster” (Hijmans & van Etten, 2015) packages.

### 2.2 PSMC

#### 2.2.1 Samples, DNA extraction, sequencing, and filtering

For the PSMC analysis, we have chosen four grouse individuals that expand the cover of the geographic range of these three species relative to the ones used in our previous study (Kozma et al., 2016). These include two willow grouse, one each from Magadan (Eastern Russia), Paxson‐South‐Central Alaska (USA), one red grouse from Yorkshire Dales National Park (Northern England), and one rock ptarmigan from southwestern Greenland. One additional willow grouse sample from Frøya (Central coast of Norway) was chosen as a control, to test whether the same demographic pattern is seen as in the previously sequenced Norwegian willow grouse sample.

DNA extraction was performed using the Qiagen DNeasy Blood & Tissue Kit^®^, and DNA quality of each individual was checked on an agarose gel and subsequently measured using Quibit^®^ Fluorometer. After library preparation with the Illumina TruSeq protocol, the samples were sequenced using an Illumina HiSeq machine to generate 125‐bp paired end reads. Quality trimming was performed using Trimmomatic v0.36 (Bolger, Lohse, & Usadel, 2014), following a four‐step procedure: (1) removing Illumina TruSeq adaptors, (2) removing leading and trailing bases with quality score <5, (3) scanning the read with a four base‐pair sliding window and cutting when the average quality per base dropped below 15, and (4) removing reads that were <50 bp after trimming.

#### 2.2.2 Assembly and analysis

Properly paired reads that passed quality control were mapped to their respective willow grouse or rock ptarmigan genome (Kozma et al., 2016) using the BWA‐MEM alignment algorithm (Li, 2013) with default settings. As no exclusively red grouse genome exists, the red grouse reads were mapped to the highly related willow grouse genome (red grouse is formally recognized as a subspecies of willow grouse). Duplicate reads were marked with Picard (http://broadinstitute.github.io/picard/), and local realignment around indels was performed with the GATK IndelRealigner tool (DePristo et al., 2011; McKenna et al., 2010). The resultant mean coverage of each individual was 26× for Magadan willow grouse, 26× for Alaska willow grouse, 38× for Frøya willow grouse, 30× for red grouse, and 30× for rock ptarmigan.

Subsequent analysis followed the same procedure as in Kozma et al. (2016). Briefly, a consensus sequence was called using the SAMTOOLS v0.1.19 suite (Li et al., 2009), utilizing the *samtools mpileup*,* bcftools,* and *vcfutils.pl* (vcf2fq) pipeline. As the ‐C50 samtools options (default in the PSMC manual) for calling consensus sequence are very stringent, especially when reads have been mapped to the genome of a related species, we produced consensus sequences without this option. For individuals with mean coverage less than 10× across 1/3 of the genome, the minimum read depth (−*d*) was set to 10, to prevent low confidence calls. Unmapped and sex chromosomes were removed from the dataset so that the PSMC analysis was carried out on the resulting autosome sequences using 30 iterations (−*N* 30), *T*_max_ (−*t*) of 10, initial mutation/recombination ratio (−*r*) of 3 and atomic time interval pattern (−*p*) of “4 + 25 × 2 + 4+6”. Mutation rate of the willow grouse and rock ptarmigan have been determined previously (willow grouse: μ = 0.299 × 10^−8^; rock ptarmigan: μ = 0.310 × 10^−8^; Kozma et al., 2016), and the red grouse was assumed to be the same as that of the willow grouse.

## 3 RESULTS

### 3.1 SDM

**Figure 1:**
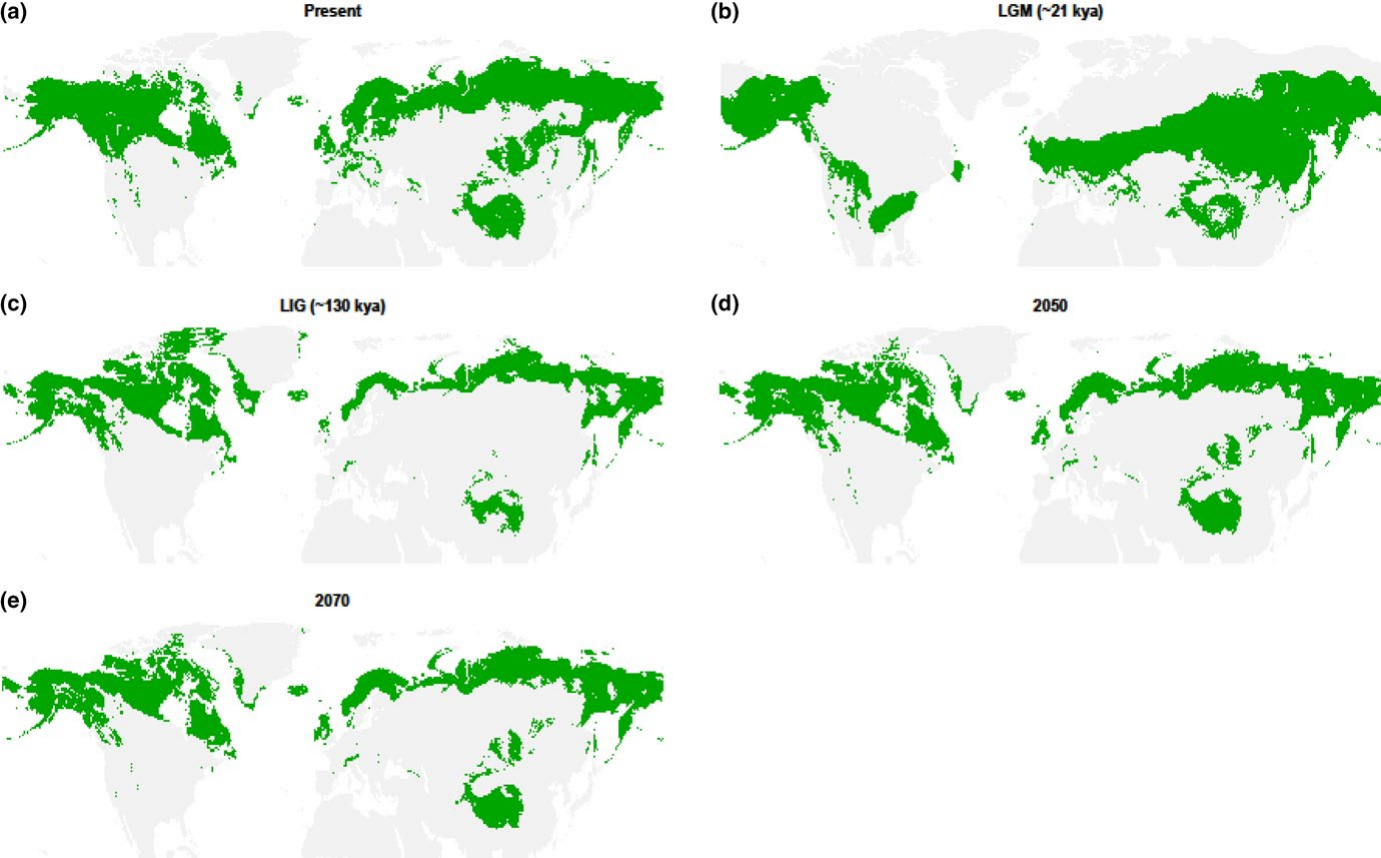
The modeled range of the willow grouse at (a) present time, (b) Last Glacial Maximum (LGM, ~21 kya), (c) Last Interglacial (LIG, ~130 kya), (d) projected year 2050, and (e) projected year 2070. Based on the GBIF dataset, with “West” subset used for model training

**Figure 2:**
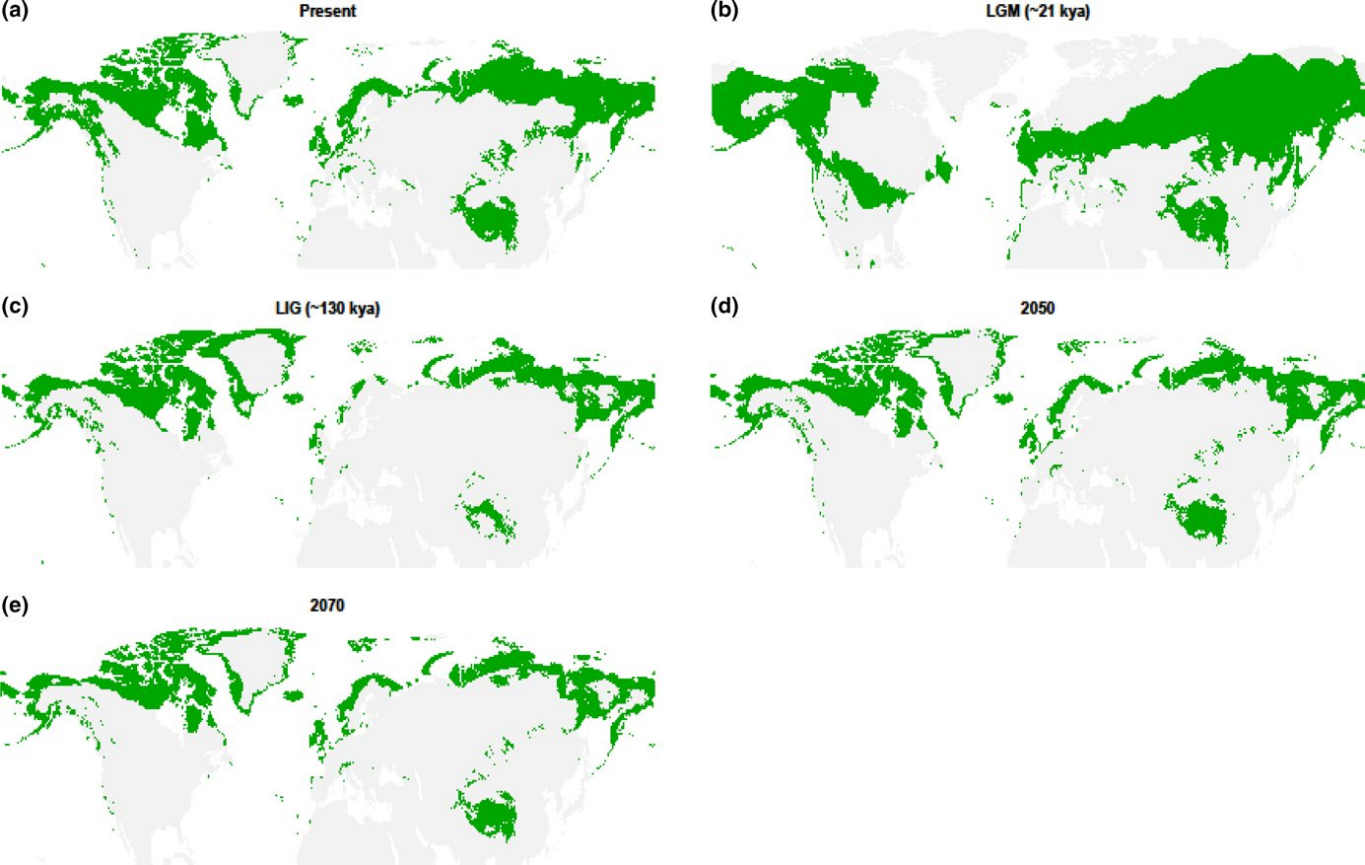
The modeled range of the rock ptarmigan at (a) present time, (b) Last Glacial Maximum (LGM, ~21 kya), (c) Last Interglacial (LIG, ~130 kya), (d) projected year 2050, and (e) projected year 2070. Based on the GBIF dataset, with “West” subset used for model training

**Figure 3:**
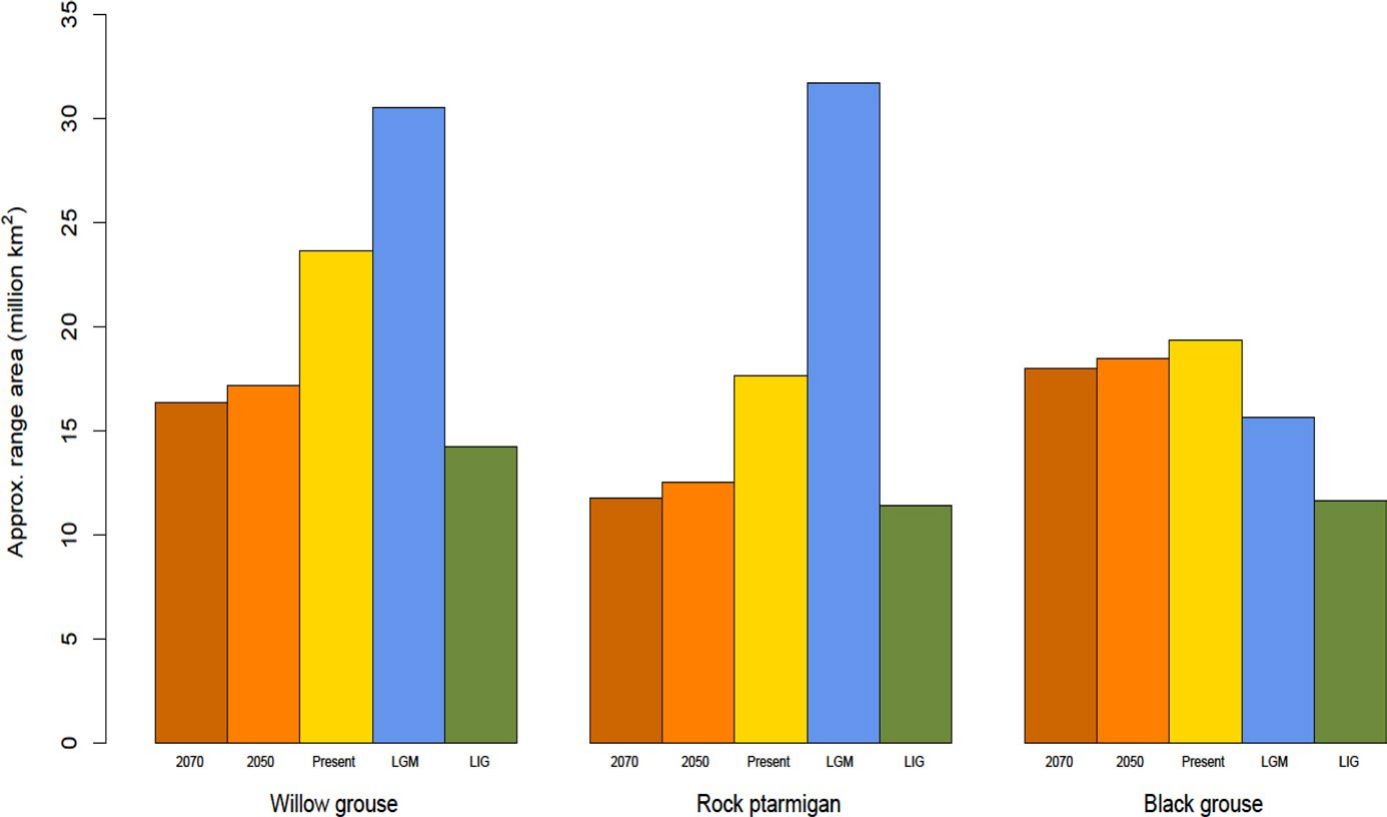
The estimated total range area of the three studied species across the modeled time periods. For the willow grouse and rock ptarmigan, the models were trained on the GBIF‐West subsets, while black grouse SDM was trained on a random subset of pseudo‐presence points in the range map dataset (see Section 2 for more detail)

For willow grouse and rock ptarmigan, geographical portioning of the GBIF dataset produced good models, where training on the “West” subset and evaluation on the “East” produced the best model (willow grouse AUC = 0.85, rock ptarmigan AUC = 0.86). These models also recovered the current range of the species (Figures 1 and 2). The same results held true for the range map dataset, where training on the “West” subset produced the best models (willow grouse AUC = 0.71, rock ptarmigan AUC = 0.81), which also recovered the current species range (Supporting Information Figures S4 and S5). Importantly, both species showed the same pattern of range size change over time across the two datasets (Figure 3 and Supporting Information Figure S7), thus corroborating the separate approaches. The warm temperatures of the LIG drastically decreased the range size of both species, with Europe maintaining only a patchy distribution and the remainder of the range being pushed into higher latitudes. Any remaining European rock ptarmigan at this stage would have been effectively cut off from the eastern population surviving in northern Siberia. For the willow grouse, the White Sea separated the European and Asian part of the range, also possibly preventing extensive gene flow. Conversely, both species experienced the largest extent of their ranges during the LGM, when the suitable habitats for both species were well connected and extended further south than the current limit. The increasing temperatures within the next century are predicted to push the species’ southern limit further north and contract the overall range to an extension similar to the one modeled during the LIG. Across Eurasia, the two species cannot move beyond the current northern limit. The willow grouse is projected to extend its northern limit in North America, with possible further stepwise colonization of Greenland. The rock ptarmigan is expected to expand its already present range in Greenland. Neither of these shifts will fully mitigate the range reduction in more southern areas, causing an overall decrease in total range size.

**Figure 4:**
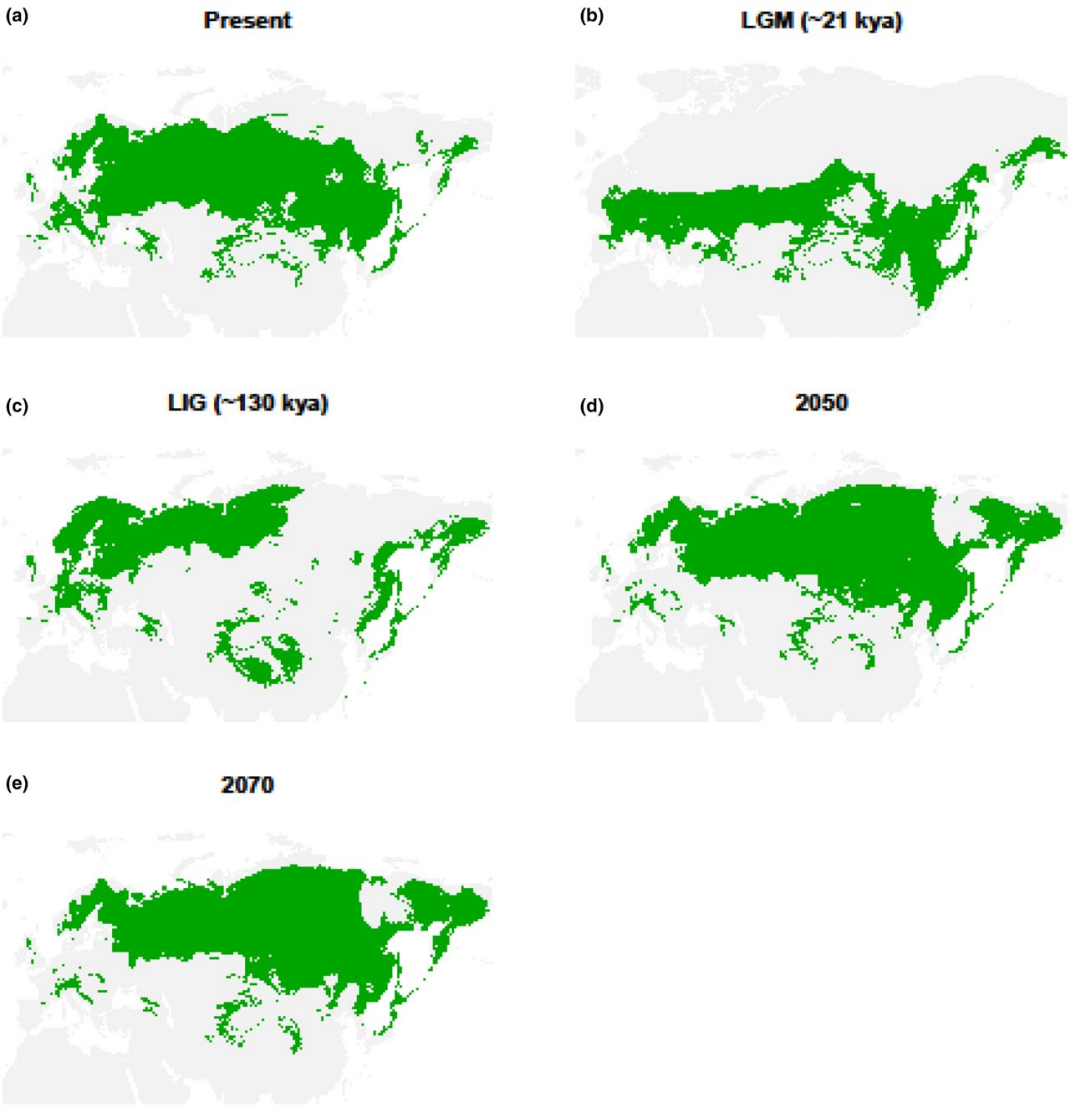
The modeled range of the black grouse at (a) present time, (b) Last Glacial Maximum (LGM, ~21 kya), (c) Last Interglacial (LIG, ~130 kya), (d) projected year 2050, and (e) projected year 2070. Based on the range map dataset, with 75% random pseudo‐presence points used for model training

For the black grouse, the GBIF dataset failed to recover the full extent of the current species range, irrespective of partitioning (Supporting Information Figure S6). This is most likely caused to the sampling bias, whereby the Asian part of the range is severely under‐represented. Moreover, the conditions in the European range are not adequate to forecast the presence of the species in the Asian range. Only random partitioning of the range map dataset produced a good model (AUC = 0.81) that also recovered the full species range (Figure 4). Because the results of the range map and GBIF datasets show good synchrony for the willow grouse and rock ptarmigan, we believe that the range map approach adequately predicts the distribution of the black grouse as well, even if the result cannot be corroborated with the GBIF dataset. For this species, the warm temperatures during the LIG also had an adverse effect on the overall range, whereby the southern limit of its extent was shifted north and the main European/West Asian region was separated from the East Asian region. The LGM saw a shift to lower latitudes, an increase in connectivity among the two regions and an overall increase in range size. This was still smaller than current range size. Lastly, the rising temperatures in the future will reduce the range size, mainly by the European habitat becoming more unsuitable and patchy. In the eastern part of its range, the species can buffer the rising temperatures by a shift to higher latitudes, in effect replacing the willow grouse and rock ptarmigan.

Overall, the results for all three species are in line with the species’ known ecologies, wherein the cold‐adapted willow grouse and rock ptarmigan have the biggest range sizes during the LGM, the more temperate black grouse has the biggest range at the present and all three had the smallest range during the LIG. Further corroborating the underlying ecologies, future warming is expected to impact the willow grouse and rock ptarmigan more severely than the black grouse. Nevertheless, the range size in all three species is expected to contract to similar levels as modeled during the LIG.

### 3.2 PSMC

**Figure 5:**
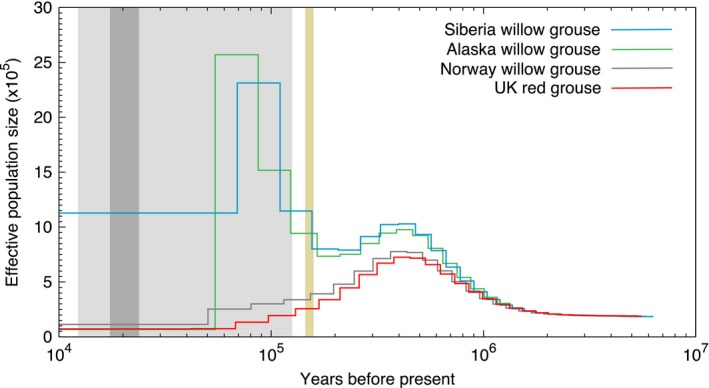
The PSMC trajectory of the willow grouse and red grouse. Yellow: LIG, light gray: last glacial period, dark gray: LGM

**Figure 6:**
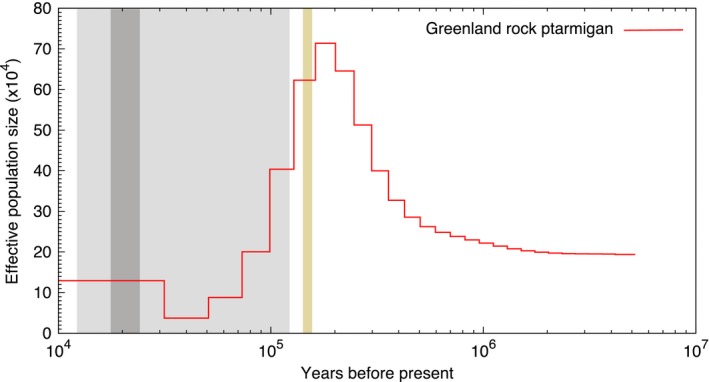
The PSMC trajectory of the Greenland rock ptarmigan. Yellow: LIG, light gray: last glacial period, dark gray: LGM

The PSMC result of the willow grouse from Norway acting as a control in this study showed the same demographic history pattern (Figure 5) as the previously published Norwegian willow grouse sample (figure 3b in Kozma et al., 2016)), supporting the overall PSMC approach. Furthermore, the PSMC of the red grouse (Figure 5) showed a highly similar pattern to that of the willow grouse, following the expectations stemming from their shared ancestry until about 6 kya. In both cases, the maximum population was reached around 400 kya, followed by a steady decrease up until 40–50 kya, from then on, the population remained stable. No increase in effective population was captured throughout the LGM. Interestingly, the willow grouse from Siberia and Alaska showed a strikingly different PSMC trajectory following the peak *N*_e_ at 400 kya (Figure 5). Both showed a second major population expansion following the onset of the last ice age (approx. 110 kya). The largest population size was not reached at LGM as the populations started to decline already from around 50–70 kya. The Alaskan willow grouse underwent a significantly larger bottleneck during the LGM than did the Siberian one. Lastly, the PSMC trajectory of the Greenland rock ptarmigan (Figure 6) showed a demographic history pattern as the previously published Icelandic rock ptarmigan (figure 3c in [17]) hinting at the shared ancestry between these two lineages. This species experienced a steady increase in *N*_e_ throughout the Pleistocene, reaching a maximum population size around 200 kya followed by a population bottleneck throughout the LGM. No population rebound following the termination of the LGM was detected in this lineage.

## 4 DISCUSSION

In this study, we have explored the demographic history of a number of grouse species using two complementary approaches: species distribution modeling to track changes in range size during different climatic conditions and coalescent‐based method to track changes in effective population size throughout these periods. Combined, the two approaches reveal a greater insight into the past than each stand‐alone method.

The PSMC is a robust method to estimate effective population size over time and here we have demonstrated its reproducibility, as a willow grouse sample from Norway shows the same pattern of *N*_e_ change as the previously published sample from the same geographic area. Moreover, the red grouse subspecies shows an identical pattern to the Norwegian sample, thus corroborating the shared history between the British red grouse and Scandinavian willow grouse (Höglund et al., 2013). We were also able to show that following the LIG, the method does indeed capture lineage‐specific population dynamics. This is seen in the divergent population size trajectories of willow grouse samples originating in Scandinavia and Britain versus Siberia and Alaska. This result further validates the observed genetic substructuring within the *L. lagopus lagopus* clade, where the Russian individuals cluster closer to the North American willow grouse (*L. lagopus muriei* and *L. lagopus alexandrae*) than to the Scandinavian willow grouse (Höglund et al., 2013). In fact, the overall similarity between the Siberian and Alaskan trajectories with the major exception being the much larger bottleneck in the Alaskan sample points to the recolonization of Alaska by the Siberian individuals during the last ice age. In the case of the rock ptarmigan, the highly similar PSMC curves illustrate the shared ancestry of the Greenland and Iceland populations, whereby a colonization of Greenland was followed by the subsequent stepwise colonization of Iceland. No population increase following the LGM was detected in the Greenland sample. The reason for this may be that the Greenland sample was sequenced at a much lower coverage than the Icelandic sample (30× vs. 101×), along with the fact that PSMC does lose power in the very recent past (Li & Durbin, 2011). It is likely that not enough loci were captured that would coalesce within this time frame, reducing the resolution of the results for this period.

Just as the PSMC approach allows the timing of delineation of separate lineages based on the underlying genomes, the SDM in turn provides the ecological background to these processes. SDM allows the tracking and quantification of change in range size, which can be compared to the fluctuations in effective population size and used to visualize the biogeographical processes that give rise to these specific lineages in the first place. In the case of the willow grouse within Eurasia, we observed that its range during the LIG was constricted to only the most northern latitudes. Large open bodies of water are enough to prevent grouse admixture, such as that between Ireland and Scotland (McMahon, Johansson, Piertney, Buckley, & Höglund, 2012) or the Aleutian Islands (Holder, Montgomerie, & Friesen, 2000). The White Sea that separates the European range from the East Asian range may have prevented extensive gene flow, giving rise to the Scandinavian and Asian lineages. While comparable species‐wide data are lacking for the rock ptarmigan, the SDM does predict similar pattern to be present in this species as well, whereby the PSMC should uncover different population size trajectories within the last ice age from Scandinavian, Siberian, and North American samples.

Overall, we see a good overlap between the SDM and PSMC methods for the Siberian willow grouse, where the SDM method predicts a steady increase in suitable habitat from the LIG up until the LGM and the PSMC does indeed show an increase in *N*_e_ which then remains at a stable high level. It is the discord between the two methods for the remaining samples that points to their distinctive underlying demographic histories. One such interesting period is the LGM. Because the climatic conditions were favorable for both willow grouse and rock ptarmigan populations to increase but the PSMC trajectories of all but the Siberian willow grouse indicate a bottleneck during this period, it reveals that the population crash must have been the result of other demographic reasons. And since the geographic regions where these samples come from were either glaciated during the LGM (Greenland, Iceland, Scandinavia and Britain; Clark et al., 2009; Clark & Mix, 2002) or were separated from the remainder of their distribution prior to the LGM (Alaska; Holder et al., 2000), these bottleneck most likely indicate the recolonization of these parts upon termination of the LGM and the creation of the Beringian land bridge, respectively. However, again we advise caution when interpreting recent changes in *N*_e_ (i.e., the plateaued lines in the PSMC plot from ~60 kya to the present) as they generally indicate the moment when PSMC loses power.

In the case of the black grouse SDM, we expected a continued increase in the number of individuals from 130 kya onwards. We also expected a much smaller population size to be present during the LIG than the LGM, as the suitable habitat during the LIG was constricted to mostly northern parts of Europe and coastal East Asia. Despite this, the previously published PSMC study indicates the opposite—a peak population size being reached around the LIG followed by a steady decrease during the glacial period, with a population recovery occurring prior the LGM (figure 3a in Kozma et al., 2016). SDM seems to support the “subdivision” hypothesis (Kozma et al., 2016), where the subdivision of the black grouse into East Asian and European subpopulations throughout the LIG artificially augments the *N*_e_ for the duration of the subdivision (see Li & Durbin, 2011 for details about the effects of subdivision on PSMC results). Upon reconnection of the two subpopulations, which occurred prior to the LGM (see the already connected range in Figure 4b), the *N*_e_ drops. Subsequently, the increase in range size prior and during the LGM can be traced in the increase in *N*_e_ starting around 30 kya. Additional sequencing of individuals from the eastern part of the black grouse distribution would be ideal to further support this hypothesis.

All three species are expected to suffer from range contractions under future climate change. The black grouse has the least amount of projected range loss, owing to its ability to expand northward into the eastern part of its distribution, but in the process displacing the willow grouse and rock ptarmigan. Remaining populations within Europe are projected to become more fragmented and isolated, which can in turn drastically affect their genetic health and overall potential for population viability (Larsson, Jansman, Segelbacher, Höglund, & Koelewijn, 2008). The situation is projected to be more serious for the cold‐adapted willow grouse and rock ptarmigan, which are both expected to lose approximately 30% of their current range. Neither can move further north within the Eurasian range and maintaining connectivity within this extent would be challenging, given their relatively short dispersal distance (~3–10 km, Berlin, Quintela, & Höglund, 2008).

There are assumptions inherent to both approaches used in this study that must be considered. For the PSMC method, the major assumption is over such long timescales, the main drivers of population change are the climatic conditions and colonization events. Grouse fall within the herbivore trophic level across their range (Martin, Doyle, Hannon, & Mueller, 2001) and have a variety of predators. The main specialist predator is the goshawk (*Accipiter gentilis*), but others include the golden eagle (*Aquila chrysaetos*), lynx (*Lynx lynx*), red fox (*Vulpes vulpes*), and wolf (*Canis lupus*; Angelstam, Lindström, & Widén, 1984; Martin et al., 2001; Pekkola, Alatalo, Pöysä, & Siitari, 2014). Furthermore, it is known that the predator–prey dynamics of grouse produce cyclical patterns of population peaks and crashes where the peaks occur periodically from three up to 11 years, depending on the geographical locations, and closely follow the cycles of the alternative prey of the predators—the hare (genus *Lepus*; summarized in Martin et al., 2001). While predators do drive the dynamics of the grouse populations over the timescale of decades, we assume that over the timescale investigated in this study the predator–prey dynamics are stable.

The key assumption made by SDMs is that species are in equilibrium with the environment and are therefore able to fill all the geographical space with suitable ecological conditions (Elith & Leathwick, 2009; Svenning et al., 2011). Even though this assumption does not hold true for all species, the analysis performed by Araújo and Pearson (2005) over groups of organisms showed that European birds are in equilibrium with climate. Niche conservatism is another important assumption to be considered when SDMs are going to be applied over long time spans (Svenning et al., 2011). While it may be hard to prove such “niche conservation” (Franklin & Miller, 2009; but also see Martinez, Peterson, & Hargrove, 2004), it is possible to use the fossil record to help judge the accuracy of the models (Lawing & Polly, 2011; Metcalf et al., 2014). For the grouse, the fossil record in Europe does agree with the models during the LGM, where these cold‐adapted species show a southward shift as well as a shift to lower altitudes (Holm & Svenning, 2014). All three species were found north of the Alps during the height of the glacial period, which is indeed captured in our model. The remoteness of the rest of the species range and the paucity of accurate fossil data over such timescale does make it hard to support the model predictions across the whole range during the LGM as well as the LIG. The assumption of niche conservatism extents into the future as well. We do acknowledge that species have the potential to change their reaction norms by phenotypic plasticity or even adaptive evolution (Jackson, Betancourt, Booth, & Gray, 2009; Merilä, 2012; Vedder, Bouwhuis, & Sheldon, 2013), but the fact that we are projecting range shift over a short period of time (~50 years) may limit the potential effects of this limitation over our model. Even considering the aforementioned limitations, we believe the models utilized in this study are sufficient to capture the large scale, species‐wide changes in range size required to contrast the PSMC patterns.

Sampling bias can also have large effects on SDMs. We do observe inadequate sampling of the black grouse in a key part of its distribution (South‐eastern Russia), and the limited known presence points are not enough to reconstruct its known range. Instead, we have relied directly on the range map of the species to build a model. This approach has been used before (Pedersen, Sandel, & Svenning, 2014; Levinsky et al., 2013) and additionally, we test it with the willow grouse and rock ptarmigan, which do not suffer from such sampling bias. In both of these species, the two models (from occurrence points and range map points) produce the same patterns in range size change across the studied time periods; therefore, we feel confident in applying this approach to the black grouse.

In this study, we have expanded the available knowledge on the demographic history of three grouse species by utilizing species distribution modeling and coalescent‐based reconstruction of past effective population size fluctuations. We have also presented the advantage of integrating approaches to overcome the limitations of single analytical methods in extrapolating meaningful conclusions from population and species modeling. In willow grouse and rock ptarmigan, we find evidence for lineage‐specific patterns of population change, with multiple recolonization events following deglaciation of its northern range. In black grouse, the two methods also hint at a potential past subdivision, but more samples need to be sequenced in order to further support this hypothesis. The species also lacks adequate occurrence data with which to build more reliable distribution models. This will have to be remedied, given the need to have a better understanding of how the current climate change will impact the demography of the species (Pacifici et al., 2015). In this regard, our models do not forecast a pretty future for the three species, as their range sizes are expected to shrink and become more fragmented, which will most likely result in overall decrease in population size and genetic diversity.

## CONFLICT OF INTEREST

None declared.

## AUTHOR CONTRIBUTIONS

R.K. and J.H. designed the study and collected the samples. R.K., M.L., B.M.B., and J‐C.S. analyzed the data. R.K. drafted the manuscript. M.L., B.M.B., J‐C.S., and J.H. improved the draft.
