# Supplementary material for: Past and potential future population dynamics of three grouse species using ecological and whole genome coalescent modeling
Source: Ecol Evol. 2018 May 29;8(13):6671–81. doi: 10.1002/ece3.4163 (PMC6053575; doi:10.1002/ece3.4163)
Supplement: Supplementary file 1 [file ECE3-8-6671-s001.docx]

**Supplementary Figures**

**Figure S1.** Correlation dendrograph. Lower numbers on the y-axis indicate higher correlations between the variables. Final set of chosen variables included: BIO5 (maximum temperature of the warmest month), BIO6 (minimum temperature of the coldest month), BIO12 (annual precipitation), BIO14 (precipitation of driest month) and BIO15 (precipitation seasonality – coefficient of variation).

**Figure S2.** GBIF presence points used for the willow grouse SDM’s. Red = GBIF-West subset, blue = GBIF-East subset.

**Figure S3.** GBIF presence points used for the rock ptarmigan SDM’s. Red = GBIF-West subset, blue = GBIF-East subset.

**Figure S4.** The modelled range of the willow grouse at a) present time, b) Last Glacial Maximum (LGM, ~21 kya), c) Last Inter-Glacial (LIG, ~130 kya), d) projected year 2050 and e) projected year 2070. Based on the range map dataset, with ‘West’ subset used for model training.

**Figure S5.** The modelled range of the rock ptarmigan at a) present time, b) Last Glacial Maximum (LGM, ~21 kya), c) Last Inter-Glacial (LIG, ~130 kya), d) projected year 2050 and e) projected year 2070. Based on the range map dataset, with ‘West’ subset used for model training.

**Figure S6.** Examples of the failure of the GBIF dataset to produce an adequate model which would recreate the black grouse’s current distribution. a) GBIF dataset with 75% random points used for training and b) GBIF-West training points – a similar geographical partitioning approach to the willow grouse and rock ptarmigan, where in this case, all the points west of the 50^th^ meridian East were used for training. Black dots indicate the presence points used in each subset. Both models fail to predict the large Asian part of the black grouse range (see Fig. 3a for correct model prediction).


**Figure S7.** The estimated total range area of the willow grouse and rock ptarmigan across the modelled time periods using the range map dataset, where the models were trained on the ‘West’ subset.
